# Cross-protective immunity of the haemagglutinin stalk domain presented on the surface of *Lactococcus lactis* against divergent influenza viruses in mice

**DOI:** 10.1080/21505594.2020.1857162

**Published:** 2020-12-29

**Authors:** Han Lei, Tong Gao, Qianhong Cen

**Affiliations:** College of Medicine, Southwest Jiaotong University, Chengdu, China

**Keywords:** *L. lactis*/pNZ8150-pgsA-HAsd, cross-protection, influenza A viruses

## Abstract

Most of the current approaches to influenza vaccine design focus on antibodies against influenza (HA). However, these influenza vaccines typically provide strain-specific protection against mostly homologous subtypes. There is an urgent need to develop a universal vaccine that confers cross-protection against influenza viruses. Of note, the HA stalk domain (HAsd) is a promising target for such an influenza vaccine. In this study, we generated recombinant *Lactococcus lactis* (*L. lactis*)/pNZ8150-phosphatidylglycerophosphate synthetase A (pgsA)-HAsd, in which pgsA was used as an anchor protein, and investigated the immunogenicity of HAsd in a mouse model by oral administration without the use of a mucosal adjuvant. Compared with *L. lactis*/pNZ8150-pgsA, mice were orally vaccinated with *L. lactis*/pNZ8150-pgsA-HAsd and then produced strong humoral and mucosal immune responses. Importantly, *L. lactis*/pNZ8150-pgsA-HAsd provided cross-protection against H5N1, H3N2 and H1N1 virus infections. Our data support the hypothesis that HAsd presented on the surface of *L. lactis* can provide cross-protective immunity against divergent influenza A viruses. Taken together, these findings suggest that *L. lactis*/pNZ8150-pgsA-HAsd can be considered an alternative approach to developing a novel universal vaccine during an influenza A pandemic.

**Abbreviations:** HA, HAsd, HA stalk domain; *L. lactis, Lactococcus lactis*; SDS-PAGE, sodium dodecyl sulfate-polyacrylamide gel electrophoresis; IFA, immunofluorescence assay; PBS, phosphate-buffered saline; pgsA, phosphatidylglycerophosphate synthetase A; SPF, specific pathogen-free; CFU, colony-forming unit; BSL-3, biosafety level-3 laboratory; TCID_50_, 50% tissue culture infective dose; ELISA, enzyme-linked immunosorbent assay; OD, optical density; LTB, liable enterotoxin B subunit; CTB, cholera toxin B subunit.

## Introduction

Influenza A viruses cause a highly infectious respiratory disease that remains a public health problem worldwide [1]. Humans can also be infected with influenza viruses that are routinely circulating in animals, such as avian influenza virus subtype A (H5N1) and swine influenza virus subtype A (H3N2 and H1N1). Vaccination is considered the most effective way to prevent and control influenza A viruses, although influenza A vaccines must be reformulated annually to match well with the predicted circulating strains [2,3]. In addition, most licensed inactivated influenza A vaccines focus on the globular head domain of the major surface glycoprotein of (HA) of the virus. These vaccines induce mainly strain-specific neutralizing antibodies, which target the highly variable regions of the globular head in the HA1 subunit. Therefore, they provide a limited breadth of protection [4,5]. In fact, antibodies against HA are the major metric by which immunity to influenza is measured, and they fall into two basic categories: globular- and stalk-directed antibodies [6]. Compared with the high mutability of the globular head region, the HA stalk domain (HAsd) is less susceptible to mutations and is relatively conserved across divergent influenza subtypes [7]. Recent studies have shown that neutralizing antibodies against the HAsd can be broadly protective in passive transfer challenge in mouse and ferret models [8–12]. Thus, the HAsd is an alternative target for a novel universal influenza vaccine because antibodies against the HAsd are capable of neutralizing diverse influenza A viruses [13].

*Lactococcus lactis* (*L. lactis*) has been engineered to express heterologous proteins [14]. Our previous studies have indicated that *L. lactis* expressing HA, HA1, neuraminidase or nucleoprotein of avian influenza H5N1 virus is a safe and effective vaccine candidate against H5N1 virus infection in mouse and chicken models with the use of a mucosal adjuvant or enteric capsule [15–18]. However, little is known regarding whether the HAsd presented on the surface of *L. lactis* in the absence of a mucosal adjuvant can provide cross-protective immunity against divergent influenza A viruses.

Thus, to investigate the immunogenicity of the HAsd presented on the surface of *L. lactis*, recombinant *L. lactis/*pNZ8150-pgsA-HAsd was constructed, in which pgsA derived from the chromosomal DNA of *Bacillus subtilis* [16], and was used as an anchor protein. Mice were orally administered recombinant *L. lactis/*pNZ8150-pgsA-HAsd, which elicited robust humoral and mucosal immune responses against influenza A viruses. Most importantly, unadjuvanted *L. lactis/*pNZ8150-pgsA-HAsd could provide cross-protective immunity against lethal challenge with homologous and heterologous influenza A viruses. These data suggest that recombinant *L. lactis/*pNZ8150-pgsA-HAsd is expected to serve as an alternative approach for a truly protective universal influenza vaccine against divergent influenza A viruses.

## Results

### *HAsd protein expressed on the surface of* L. lactis

**Figure 1.**
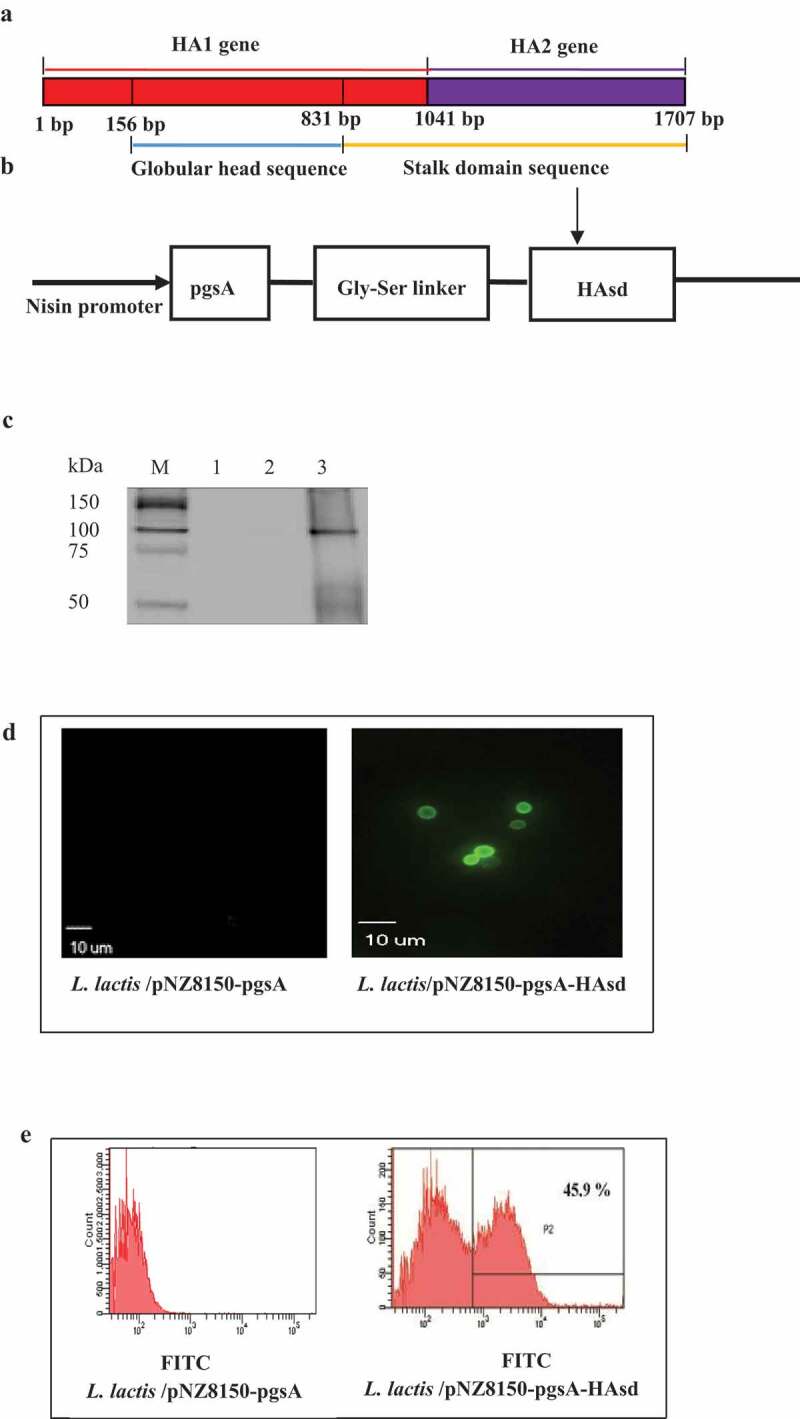
**Expression of HAsd presented on the *L. lactis* surface**. (a) Schematic description of the full length of HA gene from the H5N1 strain, containing a globular head sequence and a stalk domain sequence. Nisin promoter indicated that nisin was used as an inducer in nisin-controlled expression system. pgsA was served as an anchor protein, Gly-Ser linker was inserted between pgsA and HAsd to stabilize the fusion protein expression. (b) Schematic diagram of pNZ8150-pgsA-HAsd. A GS linker was inserted between pgsA and HAsd to stabilize HAsd protein expression. (c) Western blot analysis. Lane 1 and Lane 2: *L. lactis*/pNZ8150-pgsA; Lane 3: *L. lactis*/pNZ8150-pgsA-HAsd. (d) Immunofluorescence microscopy assay. *L. lactis*/pNZ8150-pgsA (left) and *L. lactis*/pNZ8150-pgsA- HAsd (around 0.5 μm, right) (magnification: 1,000×). (e) Flow cytometric analysis (positive rate: 45.9%)

The HAsd gene (from 831 bp to 1707 bp, 876 bp) of codon-optimized A/Vietnam/1203/2004(H5N1) (figure 1a) was cloned into the C-terminal end of pNZ8150-pgsA via a Gly-Ser linker (figure 1b). The level of HAsd protein expressed on *L. lactis* was determined by western blot analysis. A highly specific band (approximately 100 kDa) was detected for the pgsA-HAsd fusion protein in the *L. lactis*/pNZ8150-pgsA-HAsd cells (figure 1c**, lane 3**). In contrast, no band was detected in the *L. lactis*/pNZ8150-pgsA cells (figure 1c**, lanes 1 and 2).**

To confirm that HAsd protein was expressed on the surface of *L. lactis, L. lactis*/pNZ8150-pgsA and *L. lactis*/pNZ8150-pgsA-HAsd cells were examined by immunofluorescence assay (IFA) and flow cytometric analysis. There were no positive signals in the *L. lactis*/pNZ8150-pgsA cells (figure 1d and **e, left side**). By contrast, *L. lactis*/pNZ8150-pgsA-HAsd consistently exhibited specific fluorescent signals, indicating that HAsd was stably expressed on the surface of *L. lactis* (figure 1d and e, **right side**).

## Antibody responses detected by ELISA

**Figure 2.**
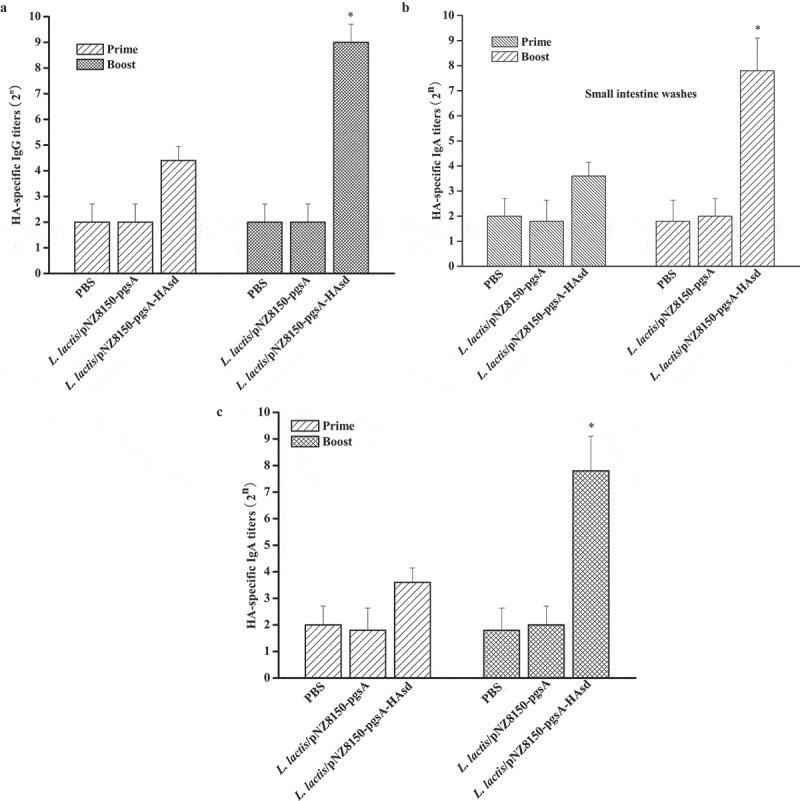
**Antibody responses detected by ELISA**. Sera and intestine and upper respiratory washes were collected from the vaccinated mice at days 15 and 34 after the initial immunization. (a) HA-specific IgG antibody responses in the sera. (b) HA-specific IgA antibody responses in the intestine washes. (c) HA-specific IgA antibody responses in the upper respiratory washes. The data are represented as the mean ± SD. Asterisks indicate significant differences compared with the PBS and *L. lactis*/pNZ8150-pgsA controls (*p* ˂ 0.05)

HA-specific antibody responses were measured by ELISA. As shown in figure 2a, there was no significant serum IgG detected in the phosphate-buffered saline (PBS), *L. lactis*/pNZ8150-pgsA or *L. lactis*/pNZ8150-pgsA-HAsd group at the prime immunization. A highly significant increase (IgG titer ≥ 2^5^) was observed in the *L. lactis*/pNZ8150-pgsA-HAsd group at the boost immunization, IgG titer induced by *L. lactis*/pNZ8150-pgsA-HAsd reach 2^8.4±0.894^, whereas there were still no significant (IgG titer <2^5^) changes in the PBS or *L. lactis*/pNZ8150-pgsA group.

Mucosal IgA antibodies were also detected in the intestine and upper respiratory washes (figure 2b and c). There were no detectable IgA antibodies in the PBS, *L. lactis*/pNZ8150-pgsA or *L. lactis*/pNZ8150-pgsA-HAsd group at the prime immunization. Only *L. lactis*/pNZ8150-pgsA- HAsd could induce an elevated level of IgA antibodies after the boost immunization, IgA titers in the intestine and upper respiratory washes were 2^7.8±1.30^ and 2^7.4±1.14^, respectively.

Collectively, these results demonstrated that mice administered *L. lactis*/pNZ8150-pgsA-HAsd orally after the prime-boost immunization could produce an elevated level of HA-specific IgG and IgA antibody responses.

## Cross-protection against virus challenge

**Figure 3.**
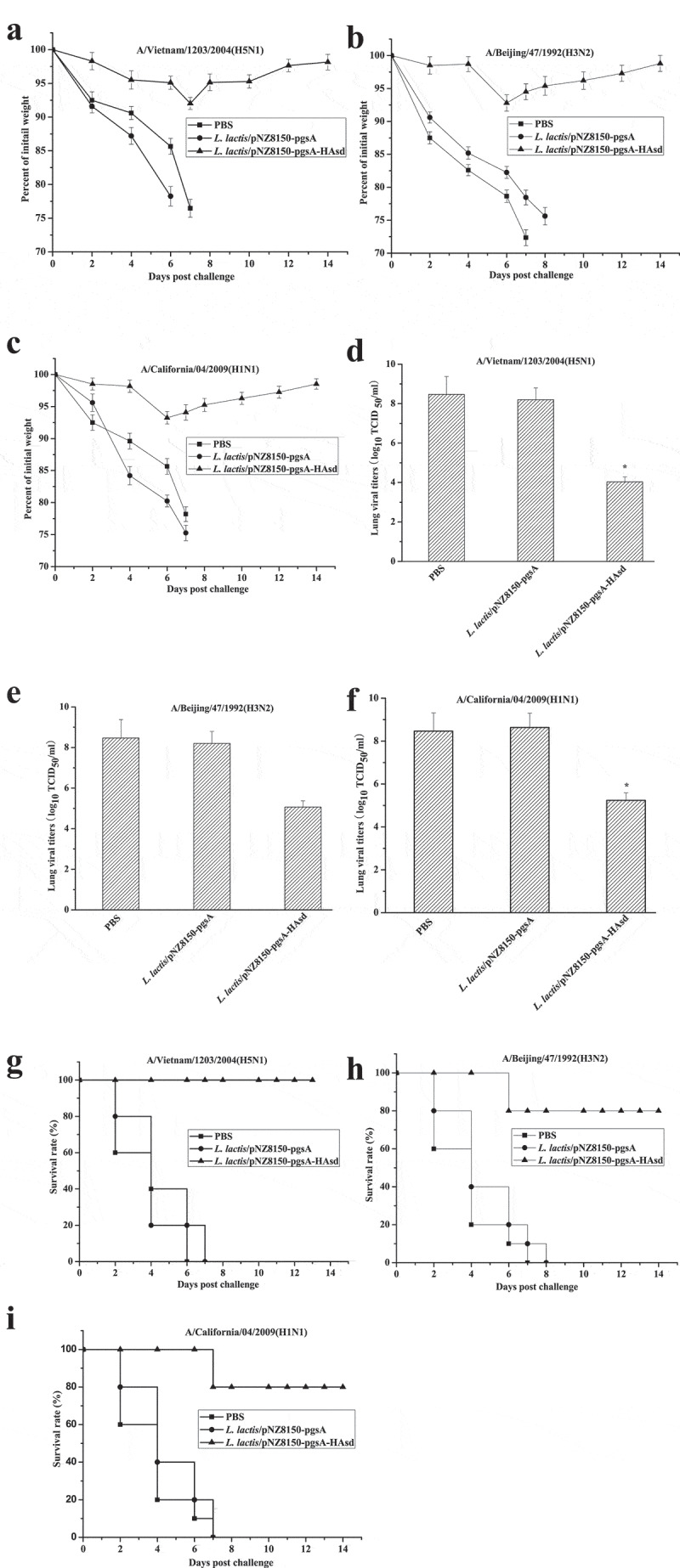
**Cross-protective efficacy against divergent influenza A viruses**. The results are expressed in terms of percent body weight **(a, b and c)**, lung virus titers **(d, e and f)** and percent survival **(g, h and i)**. Two weeks after the last immunization, mice were intranasally infected with 20 µL containing 10^4^ EID_50_ of lethal dose of A/Vietnam/1203/2004(H5N1), A/Beijing/47/1992(H3N2) or A/California/04/2009(H1N1) (C, F and I) (n = 5/group). The data for lung virus titers are represented as the mean ± SD. Asterisks indicate significant differences compared with the PBS and *L. lactis*/pNZ8150-pgsA controls (*p* ˂ 0.05)

Last, to assess the cross-protective efficacy of *L. lactis*/pNZ8150-pgsA-HAsd, all vaccinated mice were challenged with 20 µL of 10^4^ EID_50_ of A/Vietnam/1203/2004(H5N1), A/Beijing/47/1992(H3N2) or A/California/04/2009(H1N1) and monitored for 14 days. As shown in figure 3, mice administrated orally with PBS or *L. lactis*/pNZ8150-pgsA showed significant body weight loss and increased virus shedding in the lung and died within 8 days after challenge with the lethal dose of virus (figure 3a**,b** and c). In contrast, mice vaccinated orally with *L. lactis*/pNZ8150-pgsA-HAsd experienced only mild weight loss and recovered by 14 days (figure 3a, b and c). Furthermore, relatively lower lung virus titers were detected at 3 days post challenge (figure 3d**, E** and **F**). Importantly, *L. lactis*/pNZ8150-pgsA-HAsd could confer 100% (5/5), 80% (4/5) and 80% (4/5) protection against A/Vietnam/1203/2004(H5N1), A/Beijing/47/1992(H3N2) and A/California*/*04/2009(H1N1), respectively (figure 3g**, h** and i).

## Materials and methods

### *Construction of recombinant* L. lactis/*pNZ8150-pgsA-HAsd*

The HAsd (876 bp) gene of A/Vietnam/1203/2004(H5N1) that included part of HA1 (from 831 bp to 1041 bp) and complete HA2 gene (from 1041 bp to 1707 bp) (figure 1a) was PCR amplified from pcDNA3.1-HA (kindly provided by St. Jude Children’s Hospital, Memphis, TN, USA) using the following primers with *Nde* I or *EcoR* I sites underlined (forward primer: 5ʹ CTACATATG TCC ACC ATCATGAAATCCGAACTGGAGTAC 3ʹ, reverse primer: 5ʹ CCGGAATTC TTAGTTGCAGATGCGACACTGGA 3ʹ). The resulting *Nde* I/*EcoR* I fragment was cloned into pNZ8150-pgsA at the C-terminal end, in which pgsA was used as an anchor protein [16], and then electroporated into competent *L. lactis* NZ9000. The positive clone of *L. lactis*/pNZ8150-pgsA-HAsd was screened, and the plasmid was expressed as described previously [16]. *L. lactis*/pNZ8150-pgsA was used as a negative control for subsequent analysis.

## Western blot analysis

The expression level of HAsd displayed on the recombinant *L. lactis* surface was determined by western blot analysis as described previously [16]. Briefly, 10^6^-cell *L. lactis*/pNZ8150-pgsA-HAsd pellets were mixed with 60 µL of 6 × loading buffer, boiled for 10 minutes, subjected to sodium dodecyl sulfate (SDS)-polyacrylamide gel electrophoresis (SDS-PAGE) and transferred to a nitrocellulose membrane (Bio-Rad, Hercules, California, USA). The membrane was incubated with a 1:500-diluted monoclonal mouse anti-HA of A/Vietnam/1203/2004(H5N1) antibody (kindly provided by NIH Biodefense and Emerging Infections Research Resources Repository, Manassas, VA, USA) after blocking with 5% skim milk at room temperature for 2 h. After incubation overnight at 4°C, the membrane was incubated with affinity-purified horseradish peroxidase (HRP)-conjugated anti-mouse IgG (Sigma-Aldrich Corporation, St. Louis, MO, USA). The membrane was reacted with West Pico Chemiluminescent Substrate (Thermo Fisher Scientific Inc., Rockford, IL, USA) and imaged using a Molecular Imager ChemiDoc XRS System (Bio-Rad Laboratories, Inc., Hercules, CA, USA). Precision Plus Protein™ WesternC™ (Bio-Rad, USA) was used as a standard protein marker.

## Immunofluorescence assay (IFA)

The HAsd protein displayed on the *L. lactis* surface was by an IFA (Leica, Wetzlar, Germany). Briefly, 10^6^ *L. lactis*/pNZ8150-pgsA-HAsd cells were washed three times with sterile PBS, incubated with monoclonal mouse anti-HA antibody (kindly provided by NIH Biodefense and Emerging Infections Research Resources Repository, Manassas, VA, USA) at 4°C for 1 h, followed by FITC-conjugated goat anti-mouse IgG at 4°C for 30 minutes, and resuspended in 100 µL of sterile PBS. Finally, *L. lactis*/pNZ8150-pgsA-HAsd cells were used for the IFA. *L. lactis*/pNZ8150-pgsA cells were used as negative controls.

## Flow cytometric analysis

Flow cytometry was performed very similarly to IFA, as described above. Five hundred microliters of *L. lactis*/pNZ8150-pgsA-HAsd was analyzed by flow cytometric analysis (BD FacsCalibur, BD Bioscience, San Jose, CA, USA).

## Animals, vaccine, immunization and sample collection

Specific-pathogen-free (SPF) six-week-old female BALB/c mice were used (SLC Company, Shanghai, China) in this study and housed in cages ventilated under negative pressure with high efficiency particulate air filter.

The concentration of recombinant *L. lactis*/pNZ8150-pgsA-HAsd cells was adjusted to 10^12^ colony forming unit (CFU)/ml with sterile PBS. The mice (n = 39 per group) were vaccinated orally with 10^12^ CFU of *L. lactis*/pNZ8150-pgsA-HAsd at days 0, 1, 2, and 3 for prime immunization and boosted at day 17, 18, 19, and 20. PBS and *L. lactis*/pNZ8150-pgsA cells were used as controls.

At days 15 and 34 after the initial immunization, blood samples (n = 5 per group at day 15, n = 5 per group at day 34) and intestine and upper respiratory washes (n = 5 per group at day 15, n = 5 per group at day 34) were collected.

Two weeks after the last immunization, all vaccinated mice (n = 24 per group) were transferred into an enhanced biosafety level-3 laboratory (BSL-3) containment facility and challenged intranasally with 20 µL containing 10^4^ EID_50_ of lethal dose of A/Vietnam/1203/2004(H5N1) (n = 8 per group), A/Beijing/47/1992(H3N2) (n = 8 per group)or A/California/04/2009(H1N1) virus (n = 8 per group). The mice were monitored for 14 days, and body weight loss and survival rate post challenge were calculated.

Additionally, lungs from vaccinated mice were harvested for the detection of virus shedding at day 3 post infection (n = 3 per group). Tissue samples were homogenized and processed as described previously [15]. The virus titer in each sample was calculated by the Reed and Muench method [19] and expressed as 50% tissue culture infective dose (TCID_50_).

## Enzyme-linked immunosorbent assay (ELISA)

Antibody responses of serum IgG and IgA in the intestinal washes and upper respiratory washes were determined by ELISA using recombinant HA protein (2 µg/mL) of A/Vietnam/1203/2004(H5N1) (kindly provided by NIH Biodefense and Emerging Infections Research Resources Repository, Manassas, VA, USA) as a coating antigen, as described previously [16]. A 1:5,000 dilution of biotinylated goat anti-mouse IgG or IgA (R&D Systems, USA) was used as the secondary antibody, followed by a 1:1,000 dilution of streptavidin-conjugated alkaline phosphatase (AP) (R&D Systems, USA); 1.25 mM pNPP phosphatase was used as the substrate (MP Biomedicals, USA). The optical density (OD) was measured at 405 nm using an ELISA plate reader. The IgG or IgA titer was determined to be the lowest dilution with an OD greater than the mean OD of naïve controls plus 2 standard deviations, the OD405 nm value ≥ 0.2 was considered positive.

## Statistical Analysis

A two-tailed Student’s t-test and one-way ANOVA were used for all statistical analyses. A *p*-value of less than 0.05 was considered statistically significant.

## Discussion

Due to continuous antigen gene occurring mutation, the influenza A virus is considered to have the potential to cause the next pandemic [2]. Most of the currently available vaccines against influenza provide only strain-specific protection and require annual reformulation, as the influenza virus can escape vaccine-induced humoral immunity [19]. Therefore, there is an urgent need to develop a universal influenza vaccine that relies on the utilization of highly conserved antigenic targets, such as M2 and NP [20,21]. However, these conserved antigen epitopes are usually poorly exposed to the host immune system and, as such, are naturally weakly immunogenic [22]. Influenza A HA consists of two subunits: HA1, which forms a globular head, and HA2, which forms a stalk domain [10]. Antibodies against the HAsd can elicit broad neutralizing activity and provide cross-protection against heterologous influenza viruses [8,12,23]. Based on these previous findings, the HAsd is an attractive target for developing a novel universal influenza vaccine.

It has been hypothesized that the HAsd present on the surface of *L. lactis* may provide cross-protective immunity against divergent influenza A viruses. Generally, the HAsd, including HA2 and part of HA1, was displayed on the surface of *L. lactis*, and *L. lactis*/pNZ8150-pgsA-HAsd showed a specific binding profile that could be directly with anti-HA antibody and FITC-conjugated goat anti-mouse IgG. It was then tested by IFA and flow cytometric analysis. The binding profiles observed positively correlated with the display efficacy of HAsd, demonstrating that HAsd presented on the surface of *L. lactis* has reactive activity in vitro, which may contribute to immunogenicity in vivo.

It is generally recognized that *E. coli* heat-liable enterotoxin B subunit (LTB) and cholera toxin B subunit (CTB) are potent mucosal adjuvants and promote the induction of protective immunity. However, the adverse effects of these adjuvants in clinical trials, including diarrhea, low-grade fever, and vomiting, limit their further application [17,24]. Furthermore, *L. lactis* has an adjuvant function because of its inherent safety profile [25], and stimulates systemic and mucosal immunity in mice and humans with vaccines applied to mucosal surfaces [26,27]. Thus, we optimized the immune schedule so that *L. lactis*/pNZ8150-pgsA-HAsd could induce an adequate humoral response and mucosal response in the absence of a mucosal adjuvant. Moreover, the contributions of HA-specific antibodies for broad-spectrum protection against divergent influenza A viruses were emphasized.

Antigen epitope is crucial in designing vaccine. The HAsd designed in this study was codon-optimized for completely matching the antigen epitope with homologous H5N1 and 60% HAsd protein sequence similarity with heterologous H3N2 or H1N1, which provides the basis of cross-protective immunity. Furthermore, virus challenge is regarded as the gold standard for assessing the efficacy of an influenza vaccine. Therefore, mice administered orally with *L. lactis*/pNZ8150-pgsA-HAsd were protected completely (100%) (5/5) from homologous A/Vietnam/1203/2004(H5N1) virus challenge, as well as 80% (4/5) against heterologous H3N2 or H1N1 virus infection, and showed a reduced degree of virus shedding in the lung. These findings provide reliable evidence that *L. lactis*/pNZ8150-pgsA-HAsd is an effective vaccine candidate to induce cross-protective immunity against divergent influenza A viruses.

It has been demonstrated previously that adjuvanted *L. lactis*/pNZ8110-pgsA-HA1, in which pgsA has been used as an anchor protein, provided immune protection against homologous H5N1 virus in the mouse model [16]. In this study, pgsA was designed to display HAsd and was stable on the *L. lactis* surface. The HAsd represents a promising research target for a novel universal influenza vaccine. Notably, the HAsd presented on the surface of *L. lactis* has the potential to induce cross-protective immunity against divergent influenza A viruses. Thus, this study strongly demonstrates that the discovery of *L. lactis*/pNZ8150-pgsA-HAsd may allow for an alternative design of influenza vaccines that would afford broad coverage.
